# 

**Published:** 1833-10-01

**Authors:** 


### BIBLIOGRAPHICAL RECORD;

OR,

*Works received for Review since the last Quarter.*

1. The Homœopathic Medical Doctrine, or Organon of the Healing Art; a new system of Physic, translated from the German of S. HAHNEMAN. By C. H. DEVRIENT, Esq. with Notes by SAMUEL STRATTEN, M.D. 8vo. pp. 332, Dublin and London, July, 1833.

2. The Teeth, in relation to Beauty, Voice, and Health; being the result of Twenty Years' practical experience and assiduous study to produce the full development and perfect regularity of those essential Organs. By JOHN NICHOLLES, Surgeon-Dentist. 8vo. pp. 134, 1833.

3. Symptome der Ascalichen Cholera, in November and December, Berlin, &c. By Dr. ROBERT FRORIEP. 4to. pp. 89, with numerous plates. Weimar, 1832.

☞ *We return the author many thanks for this interesting work.*

4. Theoretisch-practisches Handbuch der Ceburtshulfe, &c. By LUDV. FRED. V. FRORIEP, M.D. &c. Weimar, 1832.

☞ *This is an able and erudite Vade-Mecum, theoretical and practical.*

5. Letter on the Cholera Asphyxia, now prevailing in the City of New York, addressed to James Bond Read, Chairman of the Medical Board, Savanah. By JOHN W. FRANCIS, M.D. New York, 1832.

☞ *A sensible letter; but now uninteresting.*

6. Lecture delivered in the Jeffersonian Medical College, Philadelphia, on the question, "has the parotid gland ever been extirpated?" By GRANVILLE SHARPE PATTISON, M.D. &c. 8vo. pp. 16, Philadelphia, 1833.

☞ *Dr. Pattison takes the affirmative side of the question, and we are a little surprised that the negative has been boldly affirmed by a surgical Professor in America. The present lecture is an answer, and we think a refutation of the above-mentioned assertion.*

---

7. Catalogue of Preparations, &c. in Morbid, Natural, and Comparative Anatomy, contained in the Museum of the Army Medical Department, Fort Pitt, Chatham. 8vo. pp. 266.

☞ *This catalogue is well arranged, and, as short histories of cases accompany the pathological specimen, the work combines incalculable advantages for those who visit the splendid Museum at Fort Pitt.*

---

8. An Essay on the Comparative Merits of Artificial and Natural Classification, as applied to Diseases of the Skin. By JOHN PAGET, MD. formerly President of the Royal Medical Society. 8vo. pp. 52. Reprinted from the Edinburgh Journal, No. 115.

☞ *This is a very clever performance and may be perused in our esteemed contemporary of the North, with great advantage.*

---

9. A Treatise on the Composition and Medical Properties of the Mineral Waters of Buxton, Matlock, Tunbridge Wells, Bath, &c. with instructive observations on the drinking of the Waters, &c. By Sir CHARLES SCUDAMORE, M.D. 2d. edit. corrected and enlarged. 8vo. pp. 215. 1833.

---

10. Principles and Practice of Obstetric Medicine, with Dissertations on the Diseases of Women and Children, &c. Part XXI. By DAVID D. DAVIS, M.D. 1833.

☞ *Nymphomania is still the subject of this part, and it is very ably written.*

---

11. Principles and Illustrations of Morbid Anatomy, adapted to the elements of M. ANDRAL, &c. By S. HOPE, M.D. Part VI. August, 1833.

---

12. Essays, 1st. On the Anatomy, Physiology, and Pathology of the great Sympathetic Nerve. By Mr. JAMES WILKES. 2dly. On the Anatomy of Inguinal Hernia. By Mr. W. HAMMOND, to whom were adjudged the prizes of the Birmingham School of Medicine and Surgery, for the Year 1832. 8vo. pp. 43, and 22. 1833.

---

13. A Treatise on those Disorders of the Brain and Nervous System which are usually considered and called *Mental*. By DAVID UWINS, M.D. 8vo. pp. 234. Renshaw and Rush, 1833.

---

14. Observations on Injuries and Diseases of the Rectum. By HERBERT MAYO, F.R S. Surgeon to the Middlesex Hospital. 8vo. pp. 220. Burgess and Hill, 1833.

---

15. Important Information. The Insecurity of Sir H. DAVY's Lamp, demonstrated by a series of Chemical Experiments, &c.

☞ *This improved lamp is sold by W. and F. Upton, Queen-steet, Cheapside.*

---

16. A Report of the Method and Results of the Treatment for the Malignant Cholera, by small and frequently repeated doses of calomel; with an Inquiry into the Nature and Origin of the Complaint, &c. By JOSEPH AYRE, M.D. 8vo. pp. 167. Longman and Co. Sept. 1833.

☞ *This work came too late for notice in the present number, but we strongly recommend it to the profession.*

---

17. A New Exposition of the Functions of the Nerves. By JAMES W. EARLE. Part I. 8vo. pp. 195. Longman and Co. August, 1833.

☞ *In our next.*

---

18. Description of an Apparatus intended to facilitate the Treatment of Fractures of the Lower Extremities. By T. M. GREENHOW, Surgeon to the General Infirmary for Diseases of the Eye, Newcastle-upon-Tyne. 8vo. pp. 22, with two plates. Highley, 32, Fleet-st 1833.

☞ *Testimonials of approbation from several of the first surgeons in London are appended to this pamphlet.*

---

19. A Treatise on the Diseases of the Eye. By W. LAWRENCE, F.R.S. &c. 8vo. pp. 730. August, 1833. Churchill, London.

20. An Essay on Inflammation; being an Inquiry into the Causes, Phenomena, Treatment, and Terminations of this Condition. With a View to the Elucidation of the Proximate Cause. By PHILIP LOVELL PHILLIPS, M.D. Oxon, 8vo. pp. 153. London, 1833.

21. Treatise on Diseases of the Skin; founded on New Researches in Pathological Anatomy and Physiology. By S. RAYER, D.M.P. &c. Translated from the French. By W. B. DICKINSON, Member of the Royal College of Surgeons. 8vo. pp. 399. London, 1833.

☞ *In our next.*

22. Embryologie ou Ovologie Humaine, contenant l'Histoire Descriptive et Iconographie de l'Œuf Humain. Par ALF. A. L. VELPEAU, Chirurgien de l'Hôpital de la Pitié, &c. Accompagnée de Quinze Planches, dessinées et lithographiées par A. CHAZAL. A Paris et Londres chez, J. B. BAILLIERE. 1833.

☞ *For a Review of this Work see our next Number.*

23. Mémoires de l'Academie Royale de Médecine. Tome deuxiéme—3me et 4me Fascicules.

☞ *To be noticed.*

24. Traité Pratique des Maladies de l'Utérus et de ses Annexes, &c. Par MAD. BOIVIN et M. DUGES. Tome Second.

☞ *Alluded to in our present number. To be more fully noticed.*

### General Index to the Medico-Chirurgical Review.

As soon as the 20th Vol. of the present Series is completed, a General Index will be put to press, for the present Series and the four annual volumes that preceded it. As the Index will involve a considerable expense, all Subscribers who desire to have it, must forward their names, through their respective publishers, with an order to have it sent, when completed, through the usual channel, with the Journal. Six months will be allowed for Subscribers, and no more copies will be worked off, than those ordered in the above manner. The General Index will make a small volume of about 200 pages, price the same as a Number of the Journal.

### INDEX.

A.

Abortion, Dr. Granville on ........ 130
Abouzabel, school at ............ .. 193
Abscess of kidney ..... ........... 159
Abscess of the septum narium ...... 454
Abscess of the cerebellum .......... 509
Abscess, hepatic, Dr. Roots on...... 540
Æstrus in the human subject ..... 527
Æthmoid cells, polypus in the ...... 278
Air, fatal effects of admission into the veins ...................... 241
Alcock, Mr. death of .............. 571
Alimentary canal, hyperæmia of .... 332
Alison's, Dr. outlines of pathology .. 306
Amaurosis, Lisfranc's treatment of .. 199
Amaurosis from onanism .......... 214
Amaurosis treated with strychnia .. 260
Amaurosis, Mr. Middlemore on...... 447
Ammon, Dr. on restored vision .... 156
Amputation at the hip-joint ........ 195
Anatomy, pathological, M. Cruveilhier on ........................ 137
Anatomy, pathological, Dr. Carswell on 137
Anatomy, pathological, M. Cruveilhier 329
Anatomy, morbid, by Dr. Hope .... 329
Andes, thermal waters of the ...... 234
Andral, M. on pericarditis.......... 49
Anencephalous monstrosity, case of.. 499
Aneurism, carotid, case of ........ 448
Animal magnetism, effects of ...... 184
Antimony, large doses of, in phlebitis, 486
Anus, fissure of .................. 542
Aorta, anomaly of the arch of ...... 239
Apoplexy, French opinions on ...... 504
Apoplexy of the muscles .......... 512
Apoplexy of new-born children .... 138
Apothecaries, and their usurpations.. 286
Arsenic, Berzelius on the detection of 339
Arsenic, poisoning from the fumes of 504
Arteries, bruits of the ............ 519
Arteritis and spontaneous gangrene.. 207
Ashburner on sarsaparilla.......... 477
Asphyxia of new-born children...... 265
Atrophy of the fœtal brain ....... 510
Ayre, Dr. on cholera.............. 457

B.

Babington, Dr. on the black death .. 121
Babington, Dr. (Senr.) death of .... 161
Bacon, Lord, on medicine.......... 439
Baird, Dr. unjust treatment of...... 446
Barry, Sir David, good fortune of.... 185
Baudelocque's case of the division of the symphysis pubis ......... ... 502
Beccaria, Dr. on petechial fever .... 508
Belinaye, Mr. on smilax aspera...... 477
Bell, Sir Chas. on contracted prepuce 282
Bell, Mr. George Hamilton, on diseases of the liver ................ .. 415
Berzelius, on inorganic bodies ...... 337
Biliary abscess, case of ............ 243
Bill, factories' regulation, on the .... 181
Birmingham Eye Infirmary, report of 257
Black death, Dr. Babington on ...... 121
Blandin, M. on amputation at the hip-joint.......................... 195
Blood, analysis of the serum.... ... 502
Bloxam, Mr. case of fracture and dislocation of the femur............ 480
Blundell, Dr. on animal magnetism .. 184
Boards, medical, on the............ 180
Body, discharge of worms from the .. 168
Boivin, Madam, on the uterus ...... 522
Bouillaud, M. on scalds............ 195
Bouillaud on pneumonia .......... 225
Bouillaud on the bruits of the heart and arteries.................... 519
Brain, inflammation of the ........ 459
Brain, fœtal, atrophy of............ 510
Brain, disturbances of the.......... 341
Bright, Dr. on nymphomania ...... 468
Bristol Infirmary, strange exhibition at 267
Brown, M. R. elected a member of the French Institute................ 232
Bulimia, extraordinary case of...... 206
Burns, M. Dupuytren on .......... 72
Burns, Robert, his character........ 322
Burns and scalds, Dr. Macfarlane on.. 529
Byron, Lord, his character ........ 327

C.

Cachexy, purulent, on the.......... 213
Cæsarian operation in Germany .... 420
Carbuncle, malignant, in Italy ...... 203
Carcinoma, Dr. Carswell on........ 186
Carmichael, Mr. on phrenitis ...... 459
Carotid aneurism, Mr. Montgomery's case of........................ 448
Carswell, Dr. on pathological anatomy 137
Catarrh, suffocative, on............ 441
Catheterism, Mr. Phillips on........ 112
Cephaloma, Dr. Carswell on........ 187
Cerebellum, abscess of ............ 509
Chiappa, Dr. on pellagra .......... 507

Chlorosis, diagnosis of ............ 196
Cholera in France, mortality from .. 212
Cholera, lectures on, by Majendie .. 244
Cholera, effects of, upon the eye .... 447
Cholera, Dr. Ayre on.............. 457
Cholera, Dr. Froriep on............ 514
Cholera, state regulations in Saxe-Weimar against the ............ 513
Cholera on board the Melpomene frigate........................ 528
Cicatrization of burns ............ 77
Circulation of the blood............ 247
Clavicle, excision of .............. 501
Cloquet on the fascia transversalis .. 471
Clot-Bey, M. on the Egyptian school 193
Clot-Bey, M. on the cholera........ 222
Clot-Bey, M. on lithotomy ........ 489
Coley, Mr. on ischuria vesicalis...... 190
College of Physicians, reform of .... 566
Conium maculatum, M. Fodéré on .. 218
Convulsive disease, curious case of .. 220
Cooper, Sir A. elected a member of the French Institute ............ 232
Cornea, deposition, interlamellar of.. 448
Cornea, ulceration of.............. 448
Cornea, small-pox pustule on the.... 257
Coste, Dr. on embryology .......... 527
Cough, intermittent, cure of........ 217
Coughing, fracture of rib from ...... 441
Cowper, the poet, his character...... 323
Crampton, Mr. on dislocations...... 270
Cranium, injuries of, during parturition 211
Crocodile, circulation in the........ 247
Croton oil as a counter-irritant...... 448
Croup, Dr. Walker on ............ 363
Croup, Dr. Steinmetz on .......... 501
Croup, M. Maingault on .......... 526
Cruveilhier, M. on pathological anatomy ........................ 137
Cruveilhier's pathological anatomy .. 329
Cupping, dry, on ................ 178
Cyanosis, on .................... 223

D.

Dance, M. on some remarkable cures, 228
Darwall, Dr. death of.............. 569
Davis, Dr. D. on hysteralgia........ 162
Davis, Dr. D. on hysteria .......... 190
Davis, Dr. D. on nymphomania...... 465
Death, black, Dr. Babington on...... 121
Death from severe injuries.......... 195
Defecation, Dr. O'Beirne on........ 1
Delirium tremens, Dr. O'Beirne on .. 26
Devrient's, Mr. transl. of Hahnemann, 429
Diathesis, purulent, case of ........ 242
Dieffenbach, M. on restored vision .. 158
Diptherite, M. Maingault on........ 234
Diptherite, on the ................ 245
Diptherite, M. Gendron on ........ 528
Diseases of women, Dr. R. Lee on .. 58
Diseases of urethra................ 107
Diseases, organic, of the fœtus ...... 140
Diseases of Egypt ................ 194
Diseases of the liver .............. 415
Diseases of the muscles ............ 512
Dislocation of the shoulder, Mr. Crampton on ........................ 270
Dislocation of the hip, with fracture.. 480
Dislocation of the humerus backwards 503
Duges, M. on the uterus .......... 522
Dupuytren, his lecons orales........ 542
Dupuytren, clinical lectures of ...... 68
Dysentery, Dr. O'Beirne on ........ 22
Dyspepsia, Dr. Stokes on .......... 175
Dysphagia, treatment of............ 149

E.

Eastcott, Mr. on fatty discharge from the bowels .................... 477
Ecthyma cachecticum, case of ...... 242
Egypt, diseases of ................ 194
Egypt, calculous diseases in ........ 488
Egyptian School of Medicine........ 193
Elbow-joint, excision of............ 255
Electricity, medical, on ............ 526
Embryology, Dr. Coste on ........ 527
Emissions, seminal, on ............ 171
Emmenagogues, Dr. Graves on...... 444
Ennui, labour of.................. 185
Epidemics, natural history of ...... 485
Esquimaux, phrenological description of the ........................ 474
Esquirol on the insane ............ 352
Exhalation, aromatic, from the arm.. 215
Exposition of state of Coll. of Surgeons 565
Exstrophy of the urinary bladder .... 505
Extra-uterine pregnancy, case of .... 506
Eye, on the strumous affections of .. 258
Eye, the effects of cholera upon the.. 478
Eyeball, suppuration of the ........ 444

F.

Face, ulcerations of the............ 279
Factories Bill, on the .............. 181
Fantonelli on the itch ............ 215
Fascia transversalis, Cloquet on .... 471
Fasting, extraordinary cases of...... 204
Fatty discharges from the bowels.... 477
Fees, medical, in Turkey .......... 436
Females, examination of............ 446
Fever, M. Andral on .............. 236
Fever, petechial, in Italy .......... 508
Fever, Dr. Roots on .............. 538
Fever, Dr. Latham on ............ 544
Fishes, epidemics among .......... 485
Fissure of anus, Dupuytren on...... 542
Fleming, Mr. on disease of the nose.. 454
Fletcher, Mr. on the influence of the mind on the body .............. 341
Fodéré, M. on the conium mac. .... 218
Fœtus, organic diseases of.......... 140
Fœtus, position of the ............ 235

Fœtus, position of .................. 515
Food, new article of ............... 506
Foramen ovale, open, case of........ 212
Foramen ovale open, case of........ 242
Fractures of neck of the thigh-bone.. 83
Fracture of a rib, from coughing .... 441
Frontal sinus, Dr. Milligan on the .. 359
Froriep, Dr. manual of midwifery.... 418
Froriep, Dr. on the cholera ........ 514
Froriep on induction of labour ...... 420

G.

Gairdner, Dr. on mineral springs .... 28
Galvanism, medical ................ 503
Gangræna senilis, Dupuytren on .... 492
Gangrene, spontaneous, from arteritis, 207
Gangrene of the lungs, Bouillaud on, 209
Gardner, Mr. on purpura ........... 153
Gastritis, Dr. Stokes on ............ 175
Gastralgia chlorotic, on the ........ 220
Gastrotomy, successful case of ...... 491
Genius, infirmities of, by Mr. Madden, 316
German literature, extracts from .... 493
Glasgow infirmary, cases from ...... 249
Goitre, different sorts of ........... 498
Gonorrhœa, Mr. Russell on ........ 172
Gonorrhœa, communicated from the stomach ....................... 498
Gonorrhœa, cases of ............... 549
Gout of the testis ................ 174
Granville, Dr. illustrations of abortion 130
Graves, Dr. on various diseases ..... 145
Graves, Dr. on various diseases...... 439
Graves on hydrophobia ............. 451
Graves, Dr. on German literature ... 493
Guinea-worm, within the hip-joint .. 501
Gunshot wound, effects of, on memory 214
Guthrie, Mr. on herniæ ............ 471

H.

Hæmorrhage, uterine, Dr. R. Lee on, 67
Hæmoptoe treated with tartar-emetic, 486
Halliday, Dr. on facial neuralgia .... 421
Hallucinations, Esquirol on ........ 353
Headaches in young women ........ 145
Headache, Mr. Carmichael on ...... 464
Head, injuries of the .............. 253
Health, pilgrim of ................. 183
Health, effects of misery on ........ 342
Heart, wounds of .................. 84
Heart, the bruits of ............... 519
Heart, disease of the, Dr. Jeffreys on, 365
Heart, diseases of ................. 196
Heart, on the sounds of............. 216
Heart, polypous concretions of....... 518
Heart, abnormal cavity in........... 528
Heliotrope, the .................... 183
Helminthology, M. St. Hilaire on.... 527
Hemeralopia, Dr. Patterson on....... 266
Hepatic abscess, case of............. 540
Hepatitis, different forms of........ 416
Hermaphrodism, M. Hilaire on...... 231
Hermaphrodism, M. Dutrochet on .. 233
Hermaphrodism, extraordinary case of 237
Hernia, umbilical, case of .......... 453
Hernia with a double sac .......... 283
Hernia, on, by Mr. James .......... 368
Hernia diaphragmatica, case of...... 513
Hernia humoralis, cases of.......... 550
Herniæ, Mr. Guthrie on............. 470
Hetling, Mr. on osteo-sarcoma of jaw, 261
Highlands, salutarium in .......... 160
Hip-joint, amputation at .......... 195
Homoiopathic doctrine, of Hahnemann 429
Homoiopathy in Egypt ............. 194
Hope, Dr. on morbid anatomy ...... 329
Hooping-cough treated by tartar-emetic ........................... 244
Hospital, St. George's, cases from.... 274
Hufeland on the influenza.......... 485
Hutchinson, Dr. on croton-oil ...... 448
Hydatids, conversion of............. 201
Hydrocephalus, Dr. Graves on...... 445
Hydrophobia, case of recovery from.. 451
Hydrophthalmia, Mr. Middlemore on, 259
Hymen, imperforate, effects of ...... 190
Hypertrophy of the mammæ......... 495
Hysteralgia, on .................... 162
Hysteria, fatal, Dr. D. Davis on .... 190
Hysteria, Dr. Graves on ............ 147

I.

Imperforate hymen, effects of ...... 190
Infants, diseases of................. 361
Inflammation, granular, of the os tincæ 524
Influenza at Berlin................. 484
Injuries, cause of death after......... 195
Inorganic bodies, the analysis of..... 337
Insane, illusions of the ............. 352
Insane, confinement of the ........ 356
Insanity in Italy .................. 203
Institute, French, proceedings of .... 502
Institute of France, proceedings of .. 230
Intellect, march of the ............ 184
Iodine in caries of the vertebræ...... 266
Irish pharmacy, Mr. Phelan on...... 166
Irritable uterus, on the ............ 162
Irritable testis, on the ............. 174
Irritable brain..................... 346
Ischuria renalis, of 14 years standing, 236
Itch, bad effects from the cure of .... 361
Itch, treatment of, by chloride of lime, 215

J.

James, Mr. on herniary sacs ........ 368
Jaundice, effects of, upon vision .... 444
Jaundice, yellow vision in .......... 447
Jaw, morbid growths from the lower, 274
Jaw, upper, extirpation of.......... 281
Jaws, osteo-sarcoma of the upper and lower ........................ 261
Jeffreys, Dr. on disease of the heart.. 365

Johnson, Dr. S. character .......... 320
Johnson, Mr. H. J. on gonorrhœa.... 549

K.
Kermes mineral, in pneumonia...... 216
Kidney, abscess of ................ 159
Kidney, abscess of ................ 234
Kuhn, M. on hydatids ............ 201

L.
Labour, premature, induction of .... 202
Labour, premature, induction of .... 420
Laryngeal spasm, Dr. Walker on .... 363
Laryngotomy, case of.............. 250
Laryngotomy, two cases of ........ 540
Latham, Dr. on the duration of fever, 544
Lectures, clinical, M. Dupuytren's .. 68
Lee, Dr. R. on diseases of women.... 58
Lee, Dr. R. on uterine hæmorrhage .. 67
Leech-bites, death from............ 216
Leeches, preservation of ........... 209
Legacy left by M. Portal ........... 506
Lens, regeneration of the .......... 197
Licentiates' petition to Parliament.... 566
Liddell's translation of Esquirol's treatise ........................ 352
Lisfranc on amaurosis ............ 199
Lithotomy, M. Dupuytren on ...... 87
Lithotomy, M. Clot-Bey on ........ 489
Lithotrity, report upon ............ 503
Liver, diseases of, by Mr. G. H. Bell.. 415
Liver, mortification of the.......... 443
London physicians, memorial of the.. 192
Longevity of authors, on the........ 318
Louis, M. on pericarditis .......... 49
Lungs, gangrene of the ............ 209
Lungs, paralysis of the ............ 210
Luxation of vertebræ, M. Dupuytren on 68

M.
Maclean, Mr. on abscess of kidney ... 159
Maclean, Dr. on umbilical hernia..... 453
Macfarlane, Dr. clinical reports of ... 529
Madden, Mr. on the infirmities of genius ........................ 316
Magnetism, animal, effects of ...... 184
Majendie, lectures of, on the cholera, 245
Mammæ, hypertrophy of .......... 493
Martinet, M. on neuralgia.......... 197
Mayer, M. on the reproduction of the lens ........................ 197
Mayo, Mr. on human physiology .... 91
Mayo, Mr. on injuries and diseases of the rectum .................... 289
Measles, treated by tartar-emetic .... 244
Medical Boards, on the ............ 180
Medusa, the anatomy of............ 232
Melanosis, Dr. Williams on ........ 263
Memorial of the London physicians.. 192
Memory, loss of, after a gun-shot wound ........................ 214
Menstruation, Dr. Granville on...... 130
Mental irritation and hypochondriasis, 343
Mental indigestion, Mr. Fletcher on.. 344
Mercury, use of, in syphilis ........ 390
Mercury, effects of ................ 449
Middlemore, Mr. on the diseases of the eye.......................... 257
Midwifery, Dr. Froriep's manual .... 418
Millard, Mr. on the placenta........ 66
Milligan, Dr. on the frontal sinus.... 359
Mind, influence of, on the body .... 341
Mineral springs, Dr. Gairdner on .... 28
Mineral waters, analysis of ........ 338
Monstrosity, anencephalous ........ 499
Monstrosity, curious example of .... 246
Montgomery's, Mr. case of carotid aneurism ...................... 448
Morbus coxarius, curious case of.... 501
Mortification of the liver .......... 443
Muscles, diseases of .............. 510
Muscles, apoplexy of .............. 512

N.
Negroes, inferiority of the.......... 151
Neilson, Mr. on worms ............ 168
Nervous disturbances, on .......... 341
Neuralgia of the testis ............ 174
Neuralgia cured by animal magnetism, 184
Neuralgia, treatment of, by Martinet, 197
Neuralgia cured by quinine and morphia ........................... 217
Neuralgia, Dr. Halliday on ......... 421
Neuralgia treated with belladonna.... 503
Nimmo, Mr. on restoration of vision.. 156
Nitric acid, poisoning from ........ 546
Nose, affections of the septum ...... 454
Nux vomica in paraplegia .......... 487
Nymphomania, Dr. D. Davis on ..... 465
Nymphomania, Dr. Bright on ....... 468

O.
O'Beirne, Dr. on defecation ........ 1
O'Beirne, Dr. on dysentery ........ 22
O'Beirne, Dr. on delirium tremens .. 26
Œsophagotomy, cases of .......... 239
Ophthalmia, Egyptian, cure of...... 221
Opium, effects of, in enemata ..... 148
Oppenheim, Dr. on Turkish medicine, 435
Orchitis, Mr. Russell on............ 172
Orthopody, treatment of .......... 504
Os tincæ, ulcers of................ 522
Os tincæ, granular inflammation of .. 524
Otto, Dr. on small-pox & vaccination 481
Ovary, extirpation of.............. 242
Owen, Mr. on the placenta ........ 65

P.
Pains, spasmodic, treatment of ...... 179
Paris, insalubrity of .............. 241
Paraplegia, cured with nux vomica .. 487
Paterson, Dr. on asphyxia.......... 265

Pathology, outlines of, by Dr. Alison 306
Pellagra, Dr. Chiappa on............ 507
Penis, abscess of the .............. 559
Pericarditis, M. Louis on .......... 49
Pericarditis, M. Andral on.......... 49
Petechial fever in Italy ............ 508
Petition of Licentiates of London .... 566
Phagedænic primary syphilis........ 397
Pharyngotomy, case of ............ 251
Phelan, Mr. on Irish pharmacy...... 166
Phillips, Mr. on diseases of urethra .. 107
Phillips, Mr. reclamation of ........ 573
Phlebitis, Dr. R. Lee on............ 60
Phlegmasia dolens, Dr. R. Lee on.... 61
Phlebitis treated with tartar emetic .. 486
Phrenological character of the Esquimaux ...................... 474
Phrenological estimate of the negroes 151
Phrenological development of Dr. Spurzheim ..................... 152
Phrenology of satyriasis ... ........ 238
Physician, the qualifications of a .... 227
Physicians, London, memorial of.... 192
Physicians in Turkey ............... 433
Physiology, outlines of, by Mr. Mayo 91
Pigeaux, M. on the cardiac sounds .. 216
Piles, Mr. Mayo on................ 296
Pilgrim in pursuit of Health ........ 183
Placenta, structure of, Mr. Owen on.. 65
Placenta, structure of, Mr. Millard on 66
Placenta, adhesion of the .......... 498
Plants, the physiology of .......... 232
Pleuro-pneumonia, cases of ........ 225
Plica polonica on the............. 214
Pneumonia hypostatica, on ........ 212
Poisoning from the fumes of arsenic.. 504
Polypi, cardiac, purulent matter in .. 214
Polypi of the heart......... ...... 518
Pope's character, by Mr. Madden .... 319
Porrigo, Dr. Roots on.............. 539
Portal, legacy left by .............. 506
Portio dura, neuralgia of .......... 425
Pregnancy, extra-uterine .......... 506
Prepuce, contraction of............ 282
Press, periodical.................. 284
Provincial Association, Transactions of 358
Puerperal phlebitis, on ............ 199
Puerperal fever, pathology of ...... 200
Puerperal pathology .............. 330
Pulse, state of, in hydrocephalus .... 445
Pulse, cessation of, in both arms .... 285
Purpura hæmorrhagica ............ 444
Purpura, pathology of.............. 153
Purpura hæmorrhagica, fatal case of, 210
Purulent cachexy, on the .......... 213

Q.

Quicksilver, metallic, effects of... .. 233
Quinine, hydrocyanate of .......... 219
Quinine in strumous ophthalmia .... 258

R.

Rectum, prolapsus of.............. 217
Rectum, carcinoma of ............ 252
Rectum, on disease of, by Mr. Mayo.. 289
Rectum, laceration of.............. 280
Rectum, ulcer of.................. 290
Rectum, protrusion of ............ 292
Rectum, bleeding from ............ 294
Rectum, strictures of. ............ 299
Rectum, cancer of ................ 303
Rees', Mr. translation of Berzelius .. 337
Reform, medical. ................ 566
Respiration of aquatic insects ...... 230
Retention of urine ................ 190
Retina, ossification of the ......... 215
Rheumatism of the testis .......... 174
Rhinoplastic operation ............ 489
Ricord, M. on inoculation of syphilis.. 506
Robertson, Dr. on dry cupping...... 178
Roots, Dr. on fever and rheumatism.. 539
Roots, Dr. on porrigo ............ 539
Roots, Dr. on hepatic abscess ...... 540
Roux, M. on fatal injuries.......... 195
Russell, Mr. on the testicle.......... 169

S.

Sacchi on the goitre ..... ........ 498
Salt, adulteration of ........ ..... 232
Salutarium at Oban................ 160
Sarsaparilla, effects of.............. 478
Satyriasis, case of ................ 238
Saxe-Weimar, medical regulations in 513
Scald of mouth, Bouillaud on ...... 195
Scalds, Dr. M'Farlane on .......... 529
Scalvanti, Dr. on abscess of cerebellum ....... ................... 509
Sciatica, treatment of.............. 196
Scirrhoma, Dr. Carswell on ........ 187
Scott, Mr. on extirpation of the upper jaw ........................ 281
Scott, Sir Walter, his character .... 328
Scrofula, treatment of . .......... 203
Scrotum, enlargement of .......... 172
Seminal emissions, on ......... ... 171
Septum narium, diseases of ........ 454
Small-pox, Dr. Otto on ............ 481
Smilax aspera, use of. ............ 477
Somascetics, use of. .............. 504
Springs, mineral, Dr. Gairdner on .. 28
Spurzheim, Dr. phrenological development of ..................... 152
Staphyloma, treatment of .......... 156
Statistics, moral, of France ........ 247
Steinmetz, clinical observations .... 501
Sterility, cure of.................. 202
Stirling, Mr. surgical cases by ...... 249
Stokes, Dr. on gastritis ............ 175
Stokes, Dr. on dyspepsia .......... 175
Stratten, Dr. his notes on Hahnemann 429
Strictures, Mr. Phillips on.......... 114

Sudden death, from palsy of the lungs 210

Suffocative catarrh, on ............ 441

Surgeons, Royal College of, London.. 565

Symphysis pubis, division of........ 502

Syphilis, primary, Mr. Wallace on .. 374

Syphilis, treatment of.............. 381

Syphilis, inoculation of .. ......... 506

Syphilis, Dr. Weatherill on ........ 449

T.

Taliacotian operation, by M. Velpeau 246
Taliacotian operation, by M. Labat .. 489
Taliacotian operation, by M. Dupuytren ........................ 502
Tartar-emetic plaster, death from.... 215
Testicle, Mr. Russell on............ 169
Testis, neuralgia of the ....... .... 174
Testis, rheumatism of the .......... 174
Testis, gout of the ................ 174
Tetanus, traumatic, case of ........ 528
Thermal waters of the Andes...... . 234
Thigh-bone, neck of, fractures ...... 83
Toogood, Mr. on dislocations ...... 265
Toothache, cured by quinine........ 209
Tonospasmia, curious case of ....... 509
Tracheotomy, case of........... .. 249
Tubercles, origin of ............... 201
Turkey, the medical art in.......... 433
Turkey, poisoning in .............. 438

U.

Ulcers of the os tincæ.............. 522
Ulcers, primary syphilitic .......... 374
Ureter, example of a triple ........ 504
Urethra, disease of, Mr. Phillips on .. 107
Urinary bladder, bilobed, case of .... 235
Urinary bladder, exstrophy of ...... 505
Urine, retention of................ 190
Urine, luminous .... .............. 232
Urine, state of the, in children ...... 363
Uterine hæmorrhage, Dr. R. Lee on.. 67
Uterus, irritable, Dr. Davis on the .. 162
Uterus, bilobed, case of............ 235

V.

Vaccination in France ............ 238
Vaccination in Germany .......... 481
Vaccination, Lobstein on .......... 492
Vaccine establishment, abolition of .. 181
Varicocele, on.............. ...... 175
Variola after vaccination .......... 196
Variola and the varioloid disease .... 487
Varix of the abdominal veins........ 213
Veins, obliteration of.............. 199
Veins, capillary, inflammation of.... 214
Venereal disease, Mr. Wallace on .... 38
Venereal disease, Mr. Wallace on.... 372
Vertebræ, luxation of.............. 68
Vertebræ, carious, treated with iodine 266
Vesication, extemporaneous ....... 203
Virey, M. on the position of the fœtus 515
Vision, restoration of.............. 156
Vision, double, M. Prevost on ...... 211
Vision, effects of jaundice upon...... 444
Vision, yellow, in jaundice ........ 447

W.

Walker, Dr. on diseases of infants .. 361
Wallace, Mr. on venereal disease .... 38
Wallace, Mr. on the venereal disease.. 372
Weatherill, Dr. on syphilis and mercury ........................ 449
Williams, Dr. on melanosis ........ 263
Women, diseases of, Dr. R. Lee on .. 58
Worms, discharge of .............. 168
Worms, intestinal, on the .......... 214
Wounds of heart ................ 84
Wutzer on restored vision.......... 158

### NOTICES.

A report of some interesting cases from St. George's Hospital has been unavoidably postponed till our next number.

In our last we accidentally omitted to notice a very ingenious method of illustrating the Anatomy of Hernia, about to be given to the public, by an active and intelligent surgeon, Mr. Bloxam, of Hanover Street, Hanover Square. When it comes before us we will give a more particular account of it.

